# Supplementary material for: Impact of pregabalin reclassification as a controlled substance in Egypt on gabapentinoid and opioid utilization: A repeated cross-sectional study
Source: PLoS One. 2025 Dec 5;20(12):e0337833. doi: 10.1371/journal.pone.0337833 (PMC12680176; doi:10.1371/journal.pone.0337833)

**Figure S1: Percent change calculation and example calculation of overall gabapentinoid 3-month post-policy change**


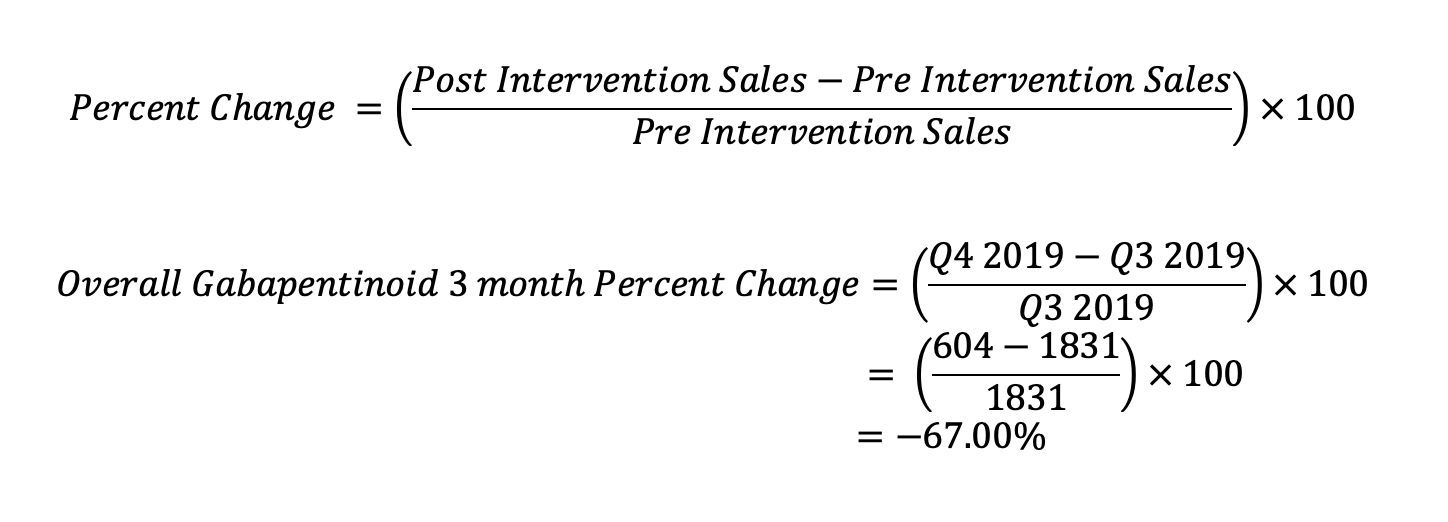

Supplement: S1 Fig — (DOCX) [file pone.0337833.s001.docx]
