# Supplementary figures and images for: Impact of pregabalin reclassification as a controlled substance in Egypt on gabapentinoid and opioid utilization: A repeated cross-sectional study
Source: PLoS One. 2025 Dec 5;20(12):e0337833. doi: 10.1371/journal.pone.0337833 (PMC12680176; doi:10.1371/journal.pone.0337833)

**Figure S2: ACF and PACF plot for overall Gabapentinoid**
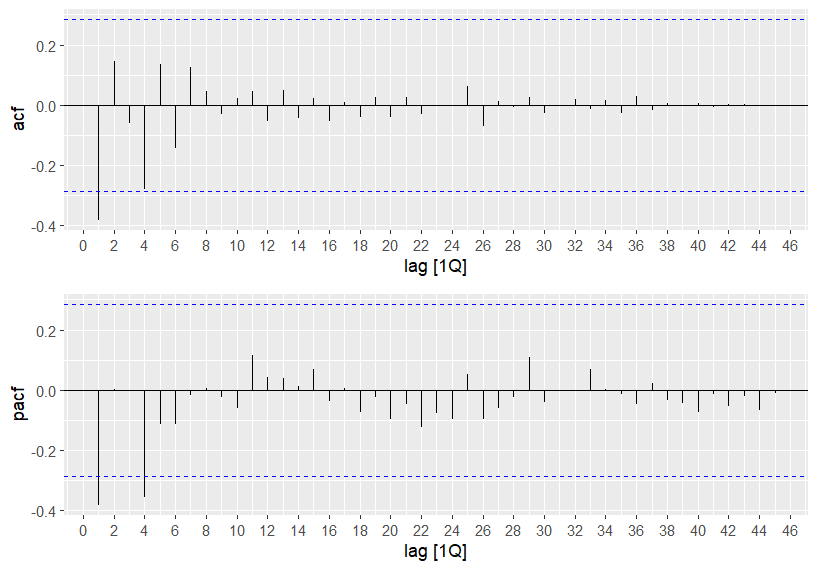

Supplement: S2 Fig — (DOCX) [file pone.0337833.s002.docx]
