# Supplementary material for: Impact of pregabalin reclassification as a controlled substance in Egypt on gabapentinoid and opioid utilization: A repeated cross-sectional study
Source: PLoS One. 2025 Dec 5;20(12):e0337833. doi: 10.1371/journal.pone.0337833 (PMC12680176; doi:10.1371/journal.pone.0337833)
Supplement: S1 Table — (DOCX) [file pone.0337833.s003.docx]

**Table S1: ARIMA model intervention coding.**

| Step | {1, year >= 2019.75; 0 elsewhere} |
| --- | --- |
| Pulse | {1, year == 2019.75; 0 elsewhere} |
| Ramp | {i, year >= 2019.75;0 elsewhere} where i is the number of periods(quarters) after third quarter of 2019. |
