# Supplementary material for: Impact of pregabalin reclassification as a controlled substance in Egypt on gabapentinoid and opioid utilization: A repeated cross-sectional study
Source: PLoS One. 2025 Dec 5;20(12):e0337833. doi: 10.1371/journal.pone.0337833 (PMC12680176; doi:10.1371/journal.pone.0337833)
Supplement: S2 Table — (DOCX) [file pone.0337833.s004.docx]

**Table S2. Kwiatkowski unit root test results.**

|  | **kpss stat** | **kpss pvalue** |
| --- | --- | --- |
| **Gabapentinoid d = 0** | 1.23 | 0.01 |
| **Gabapentinoid d = 1** | 0.0868 | 0.1 |
| **Opioid d = 0** | 0.385 | 0.0838 |
