# Supplementary material for: Impact of pregabalin reclassification as a controlled substance in Egypt on gabapentinoid and opioid utilization: A repeated cross-sectional study
Source: PLoS One. 2025 Dec 5;20(12):e0337833. doi: 10.1371/journal.pone.0337833 (PMC12680176; doi:10.1371/journal.pone.0337833)
Supplement: S3 Table — (DOCX) [file pone.0337833.s005.docx]

**Table S3. AICc and BIC fit statistics for some models considered.**

| **Drugs** | **Model Formula** | **AICc** | **BIC** |
| --- | --- | --- | --- |
| Gabapentinoid | drift + ramp + pulse + ARIMA(0,1,1) | -46.49 | -38.85 |
|  | drift + ramp + pulse + ARIMA(1,1,0) | -46.52 | -38.88 |
|  | drift + ramp + pulse + ARIMA(0,1,0)(1,0,0) | -49.20 | -41.56 |
|  | drift + ramp + pulse + ARIMA(0,1,0)(0,0,1) | -48.56 | -40.91 |
|  | **drift + ramp + pulse + ARIMA(0,1,0)(0,0,2)** | **-51.02** | **-42.20** |
| Opioid | intercept + ramp + step + ARIMA(0,0,3) | 117.65 | 127.50 |
|  | intercept + ramp + step + ARIMA(0,0,2) | 115.60 | 124.42 |
|  | intercept + ramp + step + ARIMA(0,0,1) | 115.14 | 122.78 |
|  | intercept + ramp + step + ARIMA(1,0,0) | 114.84 | 122.49 |
|  | intercept + ramp + step | 114.07 | **120.41** |
|  | intercept + ramp + step + ARIMA(1,0,0)(0,0,2) | 113.47 | 123.32 |
|  | intercept + ramp + step + ARIMA(1,0,0)(1,0,0) | 113.41 | 122.23 |
|  | **intercept + ramp + step + ARIMA(1,0,0)(1,0,2)** | **111.95** | 122.69 |
