# Supplementary material for: Impact of pregabalin reclassification as a controlled substance in Egypt on gabapentinoid and opioid utilization: A repeated cross-sectional study
Source: PLoS One. 2025 Dec 5;20(12):e0337833. doi: 10.1371/journal.pone.0337833 (PMC12680176; doi:10.1371/journal.pone.0337833)
Supplement: Attachment — Submitted filename: Response to Reviewers.docx [file pone.0337833.s007.docx]

November, 2025

Dr. Claudia Garcia Serpa Osorio-de-Castro, Ph.D

Academic Editor

PLOS ONE

Re: PLOS ONE Decision: Revision required - PONE-D-25-13595

Dear Dr. Osorio-de-Castro,

We are grateful for the suggestions provided by the reviewers and believe these comments have helped strengthen this paper. We have made the suggested changes to the manuscript based on the suggestions from the reviewer’s comments. A point-by-point response to the comments is provided below. In the manuscript with tracked changes we have highlighted all changes made since the original submission.

Sincerely,

Araniy Santhireswaran

**Reviewer 1 Comments**

The manuscript "Impact of reclassification of pregabalin as a controlled substance in Egypt: a repeated cross-sectional analysis" addresses and evaluates the impact of an important pharmaceutical policy intervention in Egypt using a quasi-experimental research design.
The authors have used proprietary IQVIA data, which is sales data, to evaluate the policy's impact. Uncontrolled ITS can be considered a reasonable research design to evaluate such policy intervention, given that the data has a sufficient number of pre- and post-intervention data points, which the study had. However, it is important to note that this approach assumes that there are no time-varying confounders, no concomitant policy changes affecting the outcome in question and a stable population. These factors should be adequately reported and discussed in the discussion section.

***Response*: Thank you for this comment. We agree that ITS analysis assumes the absence of time-varying confounders, no concurrent policy changes, and a stable population. These assumptions are inherent to the ecological nature of the study design. While no other major regulatory or policy changes occurred during the intervention period (Q3 2019), we recognize that ecological ITS designs cannot fully rule out the influence of unmeasured external factors. We have revised the discussion section to clarify the assumptions underlying our analytic approach.**

***Change*: *Additionally, this study used an ecological interrupted time series design, which evaluates aggregate-level trends in drug utilization over time rather than individual-level behavior. While this approach is robust for assessing policy impacts, it relies on several key assumptions, including a stable underlying population, no concurrent external interventions, and no time-varying confounders during the study period. Although no other major regulatory or clinical policy changes occurred at the time of the pregabalin policy implementation, it is important to acknowledge these underlying assumptions and the inherent limitations of this design.***

The authors reported that they have taken care of seasonality and non-stationarity by using differencing and selected AR and MA terms using AC and PAC plots, which should be submitted in the supplementary file.

***Response*: Thank you for pointing this out. We have included the AC and PAC plots in the supplement now as Figure S2, and the seasonal and non-seasonal AR and MA terms in tables 2 and 3 of the results.**

***Change*:**

***Table 2: ARIMA model coefficients, p-values and Ljung-Box test results for overall gabapentinoids.***

| ***Model: Intercept + Ramp + Pulse + ARIMA (0, 1, 0)(0, 0, 2)*** | | | | |
| --- | --- | --- | --- | --- |
|  | ***Coefficient*** | ***Standard Error*** | ***95% Confidence Interval*** | ***p-value*** |
| ***Intercept*** | ***0.095*** | ***0.029*** | ***0.039, 0.151*** | ***< 0.001*** |
| ***Ramp*** | ***-0.115*** | ***0.043*** | ***-0.199, -0.030*** | ***0.008*** |
| ***Pulse*** | ***-0.894*** | ***0.065*** | ***-1.022, -0.766*** | ***< 0.001*** |
| ***Seasonal MA1*** | ***-0.310*** | ***0.130*** | ***-0.565, -0.054*** | ***0.017*** |
| ***Seasonal MA2*** | ***0.739*** | ***0.355*** | ***0.043, 1.435*** | ***0.038*** |
| ***sigma^2^: 0.014*** | | | | |
| ***Ljung-Box Test: Q*= 2.991, degrees of freedom = 6, p-value = 0.810*** | | | | |

***Table 3: ARIMA model coefficients, p-values and Ljung-Box test results for overall opioids.***

| ***Model: Intercept + Ramp + Pulse + ARIMA (1, 0, 0)(1, 0, 2)*** | | | | |
| --- | --- | --- | --- | --- |
|  | ***Coefficient*** | ***Standard Error*** | ***95% Confidence Interval*** | ***p-value*** |
| ***Intercept*** | ***6.110*** | ***0.221*** | ***5.678, 6.542*** | ***< 0.001*** |
| ***Ramp*** | ***-0.055*** | ***0.049*** | ***-0.151, 0.040*** | ***0.256*** |
| ***Step*** | ***1.569*** | ***0.607*** | ***0.379, 2.759*** | ***0.010*** |
| ***Non-seasonal AR1*** | ***0.346*** | ***0.182*** | ***-0.010, 0.702*** | ***0.057*** |
| ***Seasonal AR1*** | ***-0.582*** | ***0.223*** | ***-1.019, -0.145*** | ***0.009*** |
| ***Seasonal MA1*** | ***0.463*** | ***0.229*** | ***0.015, 0.911*** | ***0.043*** |
| ***Seasonal MA2*** | ***0.643*** | ***0.258*** | ***0.138, 1.148*** | ***0.013*** |
| ***sigma^2^: 0.449*** | | | | |
| ***Ljung-Box Test: Q*= 1.495, degrees of freedom = 4, p-value = 0.828*** | | | | |

***Figure S2: ACF and PACF plot for overall Gabapentinoid***

Furthermore, the presentation of the results, especially about pre- and post-intervention magnitude changes (descriptive stats) in the table, can be improved significantly by having explicit column headings (pre- and post-intervention) to report the absolute values and % changes. Please revise this.

***Response*: Thank you for this suggestion. We have reported the absolute values and clearly labeled pre- and post-intervention headings in Table 1. We agree that this improves clarity for readers.**

***Change*:**

**Table 1: Percent changes and population adjusted sales volume per 1000 of gabapentinoid and opioid sales at 3-months, 6-months and 12-months following policy change.**

| **Drug Class** | ***Pre-Intervention Population Adjusted Sales*** | ***Post-Intervention Percent Changes (Population Adjusted Sales)*** | | |
| --- | --- | --- | --- | --- |
|  |  | **3-months** | **6-months** | **12-months** |
| **Overall Gabapentinoid** | ***1831*** | ***-67.00% (604)*** | ***-31.71% (1250)*** | ***-24.35% (1385)*** |
| **Gabapentin** | ***197*** | ***+198.49% (588)*** | ***+516.57% (1214)*** | ***+583.00% (1345)*** |
| **Pregabalin** | ***1634*** | ***-99.00% (16)*** | ***-97.81% (36)*** | ***-97.57% (40)*** |
| **Overall Opioid** | ***6.12*** | ***+8.10% (6.61)*** | ***+37.23% (8.40)*** | ***+49.47% (9.15)*** |

Similarly, a better way to report the ITS (segmented regression) is to fully report the results in a tabular format where the reader can appreciate the baseline (pre-intervention and post-intervention level and trend changes) by looking at coefficients and associated 95% CIs.

***Response*: Thank you for these suggestions, we agree that showcasing model output in tabular format will help readers. We added two additional tables showcasing the coefficients for the gabapentinoid and opioid ARIMA models, confidence intervals and p-values for the ARIMA analysis.**

***Change*:**

***Table 2: ARIMA model coefficients, p-values and Ljung-Box test results for overall gabapentinoids.***

| ***Model: Intercept + Ramp + Pulse + ARIMA (0, 1, 0)(0, 0, 2)*** | | | | |
| --- | --- | --- | --- | --- |
|  | ***Coefficient*** | ***Standard Error*** | ***95% Confidence Interval*** | ***p-value*** |
| ***Intercept*** | ***0.095*** | ***0.029*** | ***0.039, 0.151*** | ***< 0.001*** |
| ***Ramp*** | ***-0.115*** | ***0.043*** | ***-0.199, -0.030*** | ***0.008*** |
| ***Pulse*** | ***-0.894*** | ***0.065*** | ***-1.022, -0.766*** | ***< 0.001*** |
| ***Seasonal MA1*** | ***-0.310*** | ***0.130*** | ***-0.565, -0.054*** | ***0.017*** |
| ***Seasonal MA2*** | ***0.739*** | ***0.355*** | ***0.043, 1.435*** | ***0.038*** |
| ***sigma^2^: 0.014*** | | | | |
| ***Ljung-Box Test: Q*= 2.991, degrees of freedom = 6, p-value = 0.810*** | | | | |

***Table 3: ARIMA model coefficients, p-values and Ljung-Box test results for overall opioids.***

| ***Model: Intercept + Ramp + Pulse + ARIMA (1, 0, 0)(1, 0, 2)*** | | | | |
| --- | --- | --- | --- | --- |
|  | ***Coefficient*** | ***Standard Error*** | ***95% Confidence Interval*** | ***p-value*** |
| ***Intercept*** | ***6.110*** | ***0.221*** | ***5.678, 6.542*** | ***< 0.001*** |
| ***Ramp*** | ***-0.055*** | ***0.049*** | ***-0.151, 0.040*** | ***0.256*** |
| ***Step*** | ***1.569*** | ***0.607*** | ***0.379, 2.759*** | ***0.010*** |
| ***Non-seasonal AR1*** | ***0.346*** | ***0.182*** | ***-0.010, 0.702*** | ***0.057*** |
| ***Seasonal AR1*** | ***-0.582*** | ***0.223*** | ***-1.019, -0.145*** | ***0.009*** |
| ***Seasonal MA1*** | ***0.463*** | ***0.229*** | ***0.015, 0.911*** | ***0.043*** |
| ***Seasonal MA2*** | ***0.643*** | ***0.258*** | ***0.138, 1.148*** | ***0.013*** |
| ***sigma^2^: 0.449*** | | | | |
| ***Ljung-Box Test: Q*= 1.495, degrees of freedom = 4, p-value = 0.828*** | | | | |

Finally, the graph representing the trend changes should also report counterfactual scenarios by dashed lines and 95% CI for the post-intervention trend line.

***Response*: Thank you for this suggestion. We have now included the graphs with the counterfactuals as Figures 3 and 4.**

***Change*:**

***Figure 3: Overall gabapentinoid sales log per 1000 population with counterfactual scenarios by dashed lines and 95% CI for the post-intervention.***

***Author analysis based on IQVIA MIDAS® quarterly volume sales data for Egypt for the period Q1 2012 to Q3 2023, reflecting estimates of real-world activity. Copyright IQVIA. All rights reserved.***

***Figure 4: Overall opioid sales per 1000 population with counterfactual scenarios by dashed lines and 95% CI for the post-intervention.***

***Author analysis based on IQVIA MIDAS® quarterly volume sales data for Egypt for the period Q1 2012 to Q3 2023, reflecting estimates of real-world activity. Copyright IQVIA. All rights reserved.***

**Reviewer 2 Comments**

1. The term “reclassification” in the title is ambiguous. Since pregabalin was designated as a controlled substance, it would be clearer if the title explicitly reflected this.

***Response*:** **Thank you for this suggestion. We have made appropriate changes to the title to improve clarity and specify that the reclassification we mention is about pregabalin being classified as a controlled substance.**

***Change*:** **Impact of pregabalin *reclassification as a controlled substance* in Egypt on gabapentinoid and opioid utilization: a repeated cross-sectional study**

2. The title should clearly specify what the “impact” refers to. Please revise the title to indicate that the study evaluates the impact on gabapentinoid and opioid utilization in Egypt.
***Response*:** **Thank you for this suggestion. We have revised the title to specify the impact.**

***Change*:** **Impact of pregabalin reclassification as a controlled substance in Egypt *on gabapentinoid and opioid utilization*: a repeated cross-sectional study**

3. The title specifies “repeated cross-sectional analysis,” but the design is more accurately described as an interrupted time series analysis using ARIMA models.
***Response*: Thank you for this suggestion. The STROBE Checklist for reporting of observational studies states that in the title we should “Indicate the study’s design with a commonly used term in the title or the abstract”. This suggestion indicates using the design, not the analysis, in the study title. Given this, we reported the study design of a repeated cross-sectional study rather than the analysis of an interrupted time series. We acknowledge that we used the term analysis in the title rather than study, which can be misleading, so we have made that change. If you would still like us to include the analysis used, we can add that into our title as well.**

***Change***: **Impact of pregabalin reclassification as a controlled substance in Egypt on gabapentinoid and opioid utilization: a repeated cross-sectional *study***

4. In the abstract, the terms “pulse,” “ramp,” and “step” are presented from a modeling perspective. It would be clearer to replace these with interpretation-oriented terms, such as immediate short-term effect, sustained level change, or gradual long-term trend change.

***Response*:** **Thank you for this comment. We have modified these terms in the abstract.**

***Change*:** **ARIMA analyses of gabapentinoid sales showed a significant *short-term effect (pulse:* p < 0.001) and a notable *gradual long-term change (ramp:* p = 0.008). In contrast, opioids exhibited a significant *short-term sustained increase (step:* p = 0.010) but a non-significant *gradual long-term change (ramp:* p = 0.256), with sales rising by up to 49.5% at one year post-policy.**

5. The description of IQVIA’s MIDAS database is insufficient for international readers who may not be familiar with it. More detail is needed on what the database is, how it is constructed, and what types of information it provides. In particular, please explain how drug information is captured and reported in MIDAS, and clarify whether this database can be considered representative for assessing medication use in Egypt. This additional context would help readers evaluate the validity of using MIDAS data for the study objectives.

***Response*: Thank you for this comment. We have added additional information about the IQVIA MIDAS data source and how information is captured. We also added additional information about the accuracy of the data and that it captures 92.6% of sales and is a good proxy for studying drug use in Egypt.**

***Change***: ***IQVIA MIDAS reports monthly sales volume data from health systems, retail pharmacies, and major retail outlets (mass merchandisers, grocery stores, and convenience stores without pharmacies) for prescription and over-the-counter (OTC) drugs by country*… An internal validation study by IQVIA reports a precision rate of 92.6% for Egypt of *wholesaler, distributer and pharmacy audits.* This *indicates that it captures 92.6% of purchases made by drugstores wholesalers and manufacturers, suggesting sales volume is a good proxy for studying drug utilization.***

6. The criteria by which the study data were selected from the MIDAS database should be described in the Methods section. Additionally, the results of this selection should be illustrated in a flow diagram and included in the Results section.

***Response*: Thank you for this comment. The data used is purchase data and is already aggregated at the national-level and, thus, identification at the pharmacy- or patient-level was not required as part of the steps. When pulling data from the MIDAS database, we filtered solely on drug name and formulation, and we have specified the drugs we have included in our methods. We did not have multiple steps when filtering for drugs, given this, we believe that a flow diagram will not help illustrate the drug selection process. However, we have made changes to the methods where we specify what “others” included. We believe this will provide transparency about the drugs included in the analysis.**

***Change*: We used IQVIA MIDAS® quarterly volume sales data for the period Q1 2012 to Q3 2023 to analyse sales of oral and parenteral formulations of pregabalin, gabapentin and opioids (including codeine, fentanyl, hydromorphone, morphine, oxycodone, tramadol, *nalbuphine and pethidine*) in Egypt.**

7. In the Methods section, the opioids are described as “including codeine, fentanyl, hydromorphone, morphine, oxycodone, tramadol, and others.” The use of “including” and “others” makes the definition unclear. A complete and explicit list of all opioid medications analyzed should be provided to ensure clarity and reproducibility.

**Response: Thank you for pointing this out. We agree that specifying the drugs included in our study will improve clarity and reproducibility. We have specified what “others” included in the methods section.**

***Change*: We used IQVIA MIDAS® quarterly volume sales data for the period Q1 2012 to Q3 2023 to analyse sales of oral and parenteral formulations of pregabalin, gabapentin and opioids (including codeine, fentanyl, hydromorphone, morphine, oxycodone, tramadol, *nalbuphine and pethidine*) in Egypt.**

8. The description of the study model in the Methods section is not sufficiently detailed for reproducibility. More specific information on the intervention coding (step, pulse, ramp) and the final ARIMA specifications, including seasonal terms, should be provided. The ITS model should also be described in equation form, if available.

***Response***: **Thank you for this suggestion, we agree that including this information will improve reproducibility. We have added the intervention coding to the supplement in Table S1, results from the Kwiatkowski root test in Table S2, and mentioned the ARIMA specifications in Tables 2 and 3 in the manuscript.**

***Change:***

***Table S1: ARIMA model intervention coding.***

| ***Step*** | ***{1, year >= 2019.75; 0 elsewhere}*** |
| --- | --- |
| ***Pulse*** | ***{1, year == 2019.75; 0 elsewhere}*** |
| ***Ramp*** | ***{i, year >= 2019.75;0 elsewhere} where i is the number of periods(quarters) after third quarter of 2019.*** |

***Table S3. Kwiatkowski unit root test results.***

|  | ***kpss stat*** | ***kpss pvalue*** |
| --- | --- | --- |
| ***Gabapentinoid d = 0*** | ***1.23*** | ***0.01*** |
| ***Gabapentinoid d = 1*** | ***0.0868*** | ***0.1*** |
| ***Opioid d = 0*** | ***0.385*** | ***0.0838*** |

***Table 2: ARIMA model coefficients, p-values and Ljung-Box test results for overall gabapentinoids.***

| ***Model: Intercept + Ramp + Pulse + ARIMA (0, 1, 0)(0, 0, 2)*** | | | | |
| --- | --- | --- | --- | --- |
|  | ***Coefficient*** | ***Standard Error*** | ***95% Confidence Interval*** | ***p-value*** |
| ***Intercept*** | ***0.095*** | ***0.029*** | ***0.039, 0.151*** | ***< 0.001*** |
| ***Ramp*** | ***-0.115*** | ***0.043*** | ***-0.199, -0.030*** | ***0.008*** |
| ***Pulse*** | ***-0.894*** | ***0.065*** | ***-1.022, -0.766*** | ***< 0.001*** |
| ***Seasonal MA1*** | ***-0.310*** | ***0.130*** | ***-0.565, -0.054*** | ***0.017*** |
| ***Seasonal MA2*** | ***0.739*** | ***0.355*** | ***0.043, 1.435*** | ***0.038*** |
| ***sigma^2^: 0.014*** | | | | |
| ***Ljung-Box Test: Q*= 2.991, degrees of freedom = 6, p-value = 0.810*** | | | | |

***Table 3: ARIMA model coefficients, p-values and Ljung-Box test results for overall opioids.***

| ***Model: Intercept + Ramp + Pulse + ARIMA (1, 0, 0)(1, 0, 2)*** | | | | |
| --- | --- | --- | --- | --- |
|  | ***Coefficient*** | ***Standard Error*** | ***95% Confidence Interval*** | ***p-value*** |
| ***Intercept*** | ***6.110*** | ***0.221*** | ***5.678, 6.542*** | ***< 0.001*** |
| ***Ramp*** | ***-0.055*** | ***0.049*** | ***-0.151, 0.040*** | ***0.256*** |
| ***Step*** | ***1.569*** | ***0.607*** | ***0.379, 2.759*** | ***0.010*** |
| ***Non-seasonal AR1*** | ***0.346*** | ***0.182*** | ***-0.010, 0.702*** | ***0.057*** |
| ***Seasonal AR1*** | ***-0.582*** | ***0.223*** | ***-1.019, -0.145*** | ***0.009*** |
| ***Seasonal MA1*** | ***0.463*** | ***0.229*** | ***0.015, 0.911*** | ***0.043*** |
| ***Seasonal MA2*** | ***0.643*** | ***0.258*** | ***0.138, 1.148*** | ***0.013*** |
| ***sigma^2^: 0.449*** | | | | |
| ***Ljung-Box Test: Q*= 1.495, degrees of freedom = 4, p-value = 0.828*** | | | | |

9. The Methods section should explain why ARIMA modeling was chosen over other common ITS approaches, such as segmented regression. Providing the rationale for this methodological choice would help readers better understand the strengths and limitations of the analysis.

**Response: Thank you for these comments, we have added further explanations in the methods for our use of ARIMA analysis rather than other ITS approaches.**

***Change*: *Compared with other interrupted time-series methods, the ARIMA framework accounts for autocorrelation and seasonality in the outcome time series, providing more reliable estimates when there is correlation between time points. Given the seasonality and autocorrelation seen in drug sales data, the ARIMA framework is better suited for this analysis.^17^***

10. The exact calculation method for the 3-, 6-, and 12-month percent changes should be provided.

***Response*:** **Thank you for this suggestion.** **We have added a sample calculation of the percent change into the supplement (Figure S1).**

***Change*:**

***Figure S1: Percent change calculation and example calculation of overall gabapentinoid 3-month post-policy change***

11. It appears that drug utilization was estimated and modeled based on the smallest unit of consumption (e.g., one tablet, one vial, or 5 mL of liquid). It is unclear whether this approach was due to limitations of the MIDAS data or for another specific reason. A clear explanation is needed as to why analyses were not conducted using standardized dose measures (e.g., defined daily dose), and this limitation should be explicitly acknowledged, as it represents a significant constraint of the study.

***Response***: **Thank you for pointing this out. Our analysis focused on pregabalin utilization within Egypt, where the product is available in only two capsule strengths and is typically prescribed as one capsule per dose. Given this consistency, the number of standard units provides an accurate and stable measure of utilization. Standardized dose metrics such as Defined Daily Doses (DDDs) are more appropriate for cross-country or multi-drug comparisons, which were beyond the scope of this study. To make this clear to readers, we have added an explanation in the methods section.**

***Change*: *Because the analysis was restricted to a single jurisdiction and comparisons were made within drug classes, standardized units provide a reliable measure of drug use. Standardized dose metrics such as defined daily doses were not used, as the consistency in formulation strengths and dosing regimens minimizes the potential for inconsistencies in measurements.***

12. The final selected ARIMA model specifications and results should be explicitly presented in the Results section. Reporting the model orders, key coefficients, and fit statistics (e.g., AICc, BIC, Ljung–Box test) would allow readers to clearly understand the modeling choices and assess the robustness of the findings.

***Response*: Thank you for this suggestion. We have added further details into the results in tables 2 and 3 and included a table in the supplement detailing AICc, BICc (Table S3) and Ljung Box Test (Tables 2 and 3) results.**

***Change*:**

***Table 2: ARIMA model coefficients, p-values and Ljung-Box test results for overall gabapentinoids.***

| ***Model: Intercept + Ramp + Pulse + ARIMA (0, 1, 0)(0, 0, 2)*** | | | | |
| --- | --- | --- | --- | --- |
|  | ***Coefficient*** | ***Standard Error*** | ***95% Confidence Interval*** | ***p-value*** |
| ***Intercept*** | ***0.095*** | ***0.029*** | ***0.039, 0.151*** | ***< 0.001*** |
| ***Ramp*** | ***-0.115*** | ***0.043*** | ***-0.199, -0.030*** | ***0.008*** |
| ***Pulse*** | ***-0.894*** | ***0.065*** | ***-1.022, -0.766*** | ***< 0.001*** |
| ***Seasonal MA1*** | ***-0.310*** | ***0.130*** | ***-0.565, -0.054*** | ***0.017*** |
| ***Seasonal MA2*** | ***0.739*** | ***0.355*** | ***0.043, 1.435*** | ***0.038*** |
| ***sigma^2^: 0.014*** | | | | |
| ***Ljung-Box Test: Q*= 2.991, degrees of freedom = 6, p-value = 0.810*** | | | | |

***Table 3: ARIMA model coefficients, p-values and Ljung-Box test results for overall opioids.***

| ***Model: Intercept + Ramp + Pulse + ARIMA (1, 0, 0)(1, 0, 2)*** | | | | |
| --- | --- | --- | --- | --- |
|  | ***Coefficient*** | ***Standard Error*** | ***95% Confidence Interval*** | ***p-value*** |
| ***Intercept*** | ***6.110*** | ***0.221*** | ***5.678, 6.542*** | ***< 0.001*** |
| ***Ramp*** | ***-0.055*** | ***0.049*** | ***-0.151, 0.040*** | ***0.256*** |
| ***Step*** | ***1.569*** | ***0.607*** | ***0.379, 2.759*** | ***0.010*** |
| ***Non-seasonal AR1*** | ***0.346*** | ***0.182*** | ***-0.010, 0.702*** | ***0.057*** |
| ***Seasonal AR1*** | ***-0.582*** | ***0.223*** | ***-1.019, -0.145*** | ***0.009*** |
| ***Seasonal MA1*** | ***0.463*** | ***0.229*** | ***0.015, 0.911*** | ***0.043*** |
| ***Seasonal MA2*** | ***0.643*** | ***0.258*** | ***0.138, 1.148*** | ***0.013*** |
| ***sigma^2^: 0.449*** | | | | |
| ***Ljung-Box Test: Q*= 1.495, degrees of freedom = 4, p-value = 0.828*** | | | | |

***Table S3. AICc and BIC for some models considered.***

| ***Drugs*** | ***Model Formula*** | ***AICc*** | ***BIC*** |
| --- | --- | --- | --- |
| *Gabapentinoid* | *drift + ramp + pulse + ARIMA(0,1,1)* | *-46.49* | *-38.85* |
|  | *drift + ramp + pulse + ARIMA(1,1,0)* | *-46.52* | *-38.88* |
|  | *drift + ramp + pulse + ARIMA(0,1,0)(1,0,0)* | *-49.20* | *-41.56* |
|  | *drift + ramp + pulse + ARIMA(0,1,0)(0,0,1)* | *-48.56* | *-40.91* |
|  | ***drift + ramp + pulse + ARIMA(0,1,0)(0,0,2)*** | ***-51.02*** | ***-42.20*** |
| *Opioid* | *intercept + ramp + step + ARIMA(0,0,3)* | *117.65* | *127.50* |
|  | *intercept + ramp + step + ARIMA(0,0,2)* | *115.60* | *124.42* |
|  | *intercept + ramp + step + ARIMA(0,0,1)* | *115.14* | *122.78* |
|  | *intercept + ramp + step + ARIMA(1,0,0)* | *114.84* | *122.49* |
|  | *intercept + ramp + step* | *114.07* | ***120.41*** |
|  | *intercept + ramp + step + ARIMA(1,0,0)(0,0,2)* | *113.47* | *123.32* |
|  | *intercept + ramp + step + ARIMA(1,0,0)(1,0,0)* | *113.41* | *122.23* |
|  | ***intercept + ramp + step + ARIMA(1,0,0)(1,0,2)*** | ***111.95*** | *122.69* |

13. In the Results section, please also report the findings for all intervention functions consistently. For gabapentinoids, the step effect is not mentioned, and for opioids, the pulse effect is not described.

***Response*: Thank you for bringing this to our attention. For gabapentinoids, only ramp and pulse functions were used, while for opioids, only ramp and step functions were used. We can see how this was not clear in our methods; we have now clarified this in our methods and clarified this in Tables 2 and 3 in the results.**

***Change*: *Step function was used for opioids to account for immediate, sustained changes, and a pulse function was used for gabapentinoids to account for immediate, temporary changes,* reflecting whether these changes persisted or returned to pre-intervention levels.**

14. The manuscript acknowledges that external factors such as concurrent regulatory changes, clinical guidelines, or shifts in prescribing practices could have influenced drug utilization trends. However, it does not explain why these factors were not incorporated into the ARIMA models. For example, the COVID-19 pandemic and other healthcare policies in Egypt may have significantly affected drug use. Without a clear rationale for excluding these variables, it is challenging to attribute the observed changes solely to the pregabalin policy.

***Response*:** **We thank the reviewer for this thoughtful comment. No major health policy or regulatory changes coincided with the timing of the pregabalin intervention, and the onset of the COVID-19 pandemic occurred several months later (Q2 2020), beyond the immediate post-intervention period. As such, no concurrent events were expected to confound the observed change. Moreover, the ARIMA framework inherently accounts for underlying temporal fluctuations, trends, and seasonality in the outcome series. Given the model’s ability to capture autocorrelation and seasonality, and the difficulty of specifying additional external interventions, we opted not to include separate covariates for these events. We have mentioned this as a limitation of the ARIMA analysis in the discussion section.**

***Change*: *Additionally, this study used an ecological interrupted time series design, which evaluates aggregate-level trends in drug utilization over time rather than individual-level behavior. While this approach is robust for assessing policy impacts, it relies on several key assumptions, including a stable underlying population, no concurrent external interventions, and no time-varying confounders during the study period. Although no other major regulatory or clinical policy changes occurred at the time of the pregabalin policy implementation, it is important to acknowledge these underlying assumptions and the inherent limitations of this design.***

15. In the Discussion, the authors state that the observations align with evidence from other countries. However, as noted, the results in France differed from those in Egypt. A more comprehensive discussion is needed to explore the reasons behind these cross-country differences.

***Response*: Thank you for pointing this out. Our goal was to discuss what has been seen in other countries in comparison with Egypt, whether it was similar or different. We have modified the language in our discussion to better align with this. We have also discussed potential reasons why these differences are being observed.**

***Change*: These observations show *both similarities and differences compared* with evidence from other countries, and comparative analyses across diverse health systems may provide additional insights into the impact of such policies… *These cross-national differences may reflect variations in regulatory enforcement, prescribing habits, and the availability of alternative pain management options, underscoring the importance of country-specific approaches when implementing drug control policies.***

16. The statement that the lack of a significant long-term trend in opioid consumption may be attributed to existing opioid regulations or differences in prescribing practices requires a more detailed interpretation supported by references.

**Response: Thank you for pointing this out. This was an oversight on our part. We have added appropriate references supporting limitations in opioid prescribing, and further explained this thought.**

**Change: However, the lack of a significant change in long-term trends in opioid consumption indicates that the policy did not lead to sustained increases. *This may be attributed to existing opioid regulations or differences in prescribing practices, such as The Egyptian Narcotics Control Law that limits morphine prescribing.^21,22^***
